# Acute exudative polymorphous vitelliform maculopathy during pembrolizumab treatment for metastatic melanoma: a case report

**DOI:** 10.1186/s12886-021-02011-4

**Published:** 2021-06-05

**Authors:** Ine Lambert, Giuseppe Fasolino, Gil Awada, Robert Kuijpers, Marcel ten Tusscher, Bart Neyns

**Affiliations:** 1 Department of Ophthalmology, Universitair Ziekenhuis Brussel, Vrije Universiteit Brussel, Laarbeeklaan 101, 1090 Jette, Belgium; 2 Department of Medical Oncology, Universitair Ziekenhuis Brussel, Vrije Universiteit Brussel, Jette, Belgium; 3 Department of Ophthalmology, Schweitzer Hospital, Dordrecht, The Netherlands

**Keywords:** Pembrolizumab, Immune checkpoint inhibitors, Acute exudative polymorphous vitelliform maculopathy, Immune related adverse event

## Abstract

**Background:**

The use of immunomodulating therapy to treat various cancers has been on the rise and these immune checkpoint inhibitors are known to cause ocular side effects. In this article a case of acute exudative polymorphous vitelliform maculopathy (AEPVM) is reported which developed during a first line treatment with pembrolizumab.

**Case presentation:**

A 54-year-old woman was referred because of blurry vision in both eyes with a yellow spot in the central visual field of the left eye. These symptoms started after four treatments with pembrolizumab (a monoclonal antibody against the programmed cell death receptor-1) for a metastatic recurrent vaginal mucosal melanoma. Her best corrected visual acuity was 10/10 in both eyes with a correction of + 2.00 bilaterally. There were no inflammatory findings in the anterior segment or the vitreous. Fundoscopy revealed an attenuation of the foveal reflex with subtle yellow-white subretinal macular deposits (vitelliform lesions) in both eyes. Fluorescein angiography did not show staining or leakage in the mid-phase, neither a late staining. Spectral-domain optical coherence tomography of the macula illustrated bilateral neurosensory retinal detachment with a thick, highly reflective band at the outer photoreceptor segment. En face structural OCT at the level of the photoreceptors showed focal areas of increased signal corresponding to hyperreflective vitelliform material. The treatment with pembrolizumab was ceased immediately. During the following visits we slowly saw an improvement of the neurosensory retinal detachment. After almost four months a total resolution of the subretinal fluid was visualized in both eyes without the use of additional treatment, though the vitelliform deposits persisted.

**Conclusions:**

The development of AEPVM in melanoma patients could be triggered by treatment with Pembrolizumab. Pembrolizumab has the potential to disturb indirectly the retinal pigment epithelium homeostasis with accumulation of lipofuscin deposits and subretinal fluid, both signs of AEPVM.

## Background

Acute exudative polymorphous vitelliform maculopathy (AEPVM) represents a rare retinal disorder characterized by multifocal round or crescent-shaped yellow subretinal deposits with a vitelliform appearance that correspond to serous retinal detachments on optical coherence tomography (OCT). AEPVM has been described as a feature of a paraneoplastic syndrome in association with different types of melanoma and carcinoma. It has also been reported with infectious diseases or as an idiopathic process. The real mechanism of disease is not completely understood [1].

In recent years the use of immunomodulating therapy to treat various cancers has been on the rise. Pembrolizumab, a commonly used immune checkpoint inhibitor, is a humanized monoclonal antibody that blocks the programmed cell death receptor-1 (PD-1) protein, which is expressed on activated T cells, and is used for the treatment of advanced, unresectable or metastatic malignant melanoma and other cancers [2]. Cancer cells express PD-L1 (programmed cell death protein ligand-1), which is a ligand for PD-1. Upon binding of PD-1 and PD-L1, activated T cells become inactive and cancer cells start to proliferate [3]. By blocking PD-1, pembrolizumab releases the inhibition on T cells and increases the immune system’s ability to attack melanoma cells and tumors. Patients receiving checkpoint inhibitors may develop immune-related adverse events (IRAEs). The most common autoimmune-like side effects are pneumonitis, colitis, hepatitis and endocrinopathies [2].

Ophthalmic side effects most frequently manifest as dry eye (< 1–5 %) and uveitis (< 1 %) (Vogt-Koyanagi-Harada disease-like uveitis). Other side effects include myasthenia gravis, inflammatory orbitopathy, keratitis, corneal ulceration and perforation, cranial nerve palsy, optic neuropathy, serous retinal detachment and neuroretinitis [4, 5].

In this article a case of AEPVM is reported that developed during a first-line treatment with pembrolizumab for a metastatic recurrent vaginal mucosal melanoma. To our best knowledge this is the first case of AEPVM during pembrolizumab monotherapy. A literature review yielded no similar cases.

## Case presentation

**Fig. 1:**
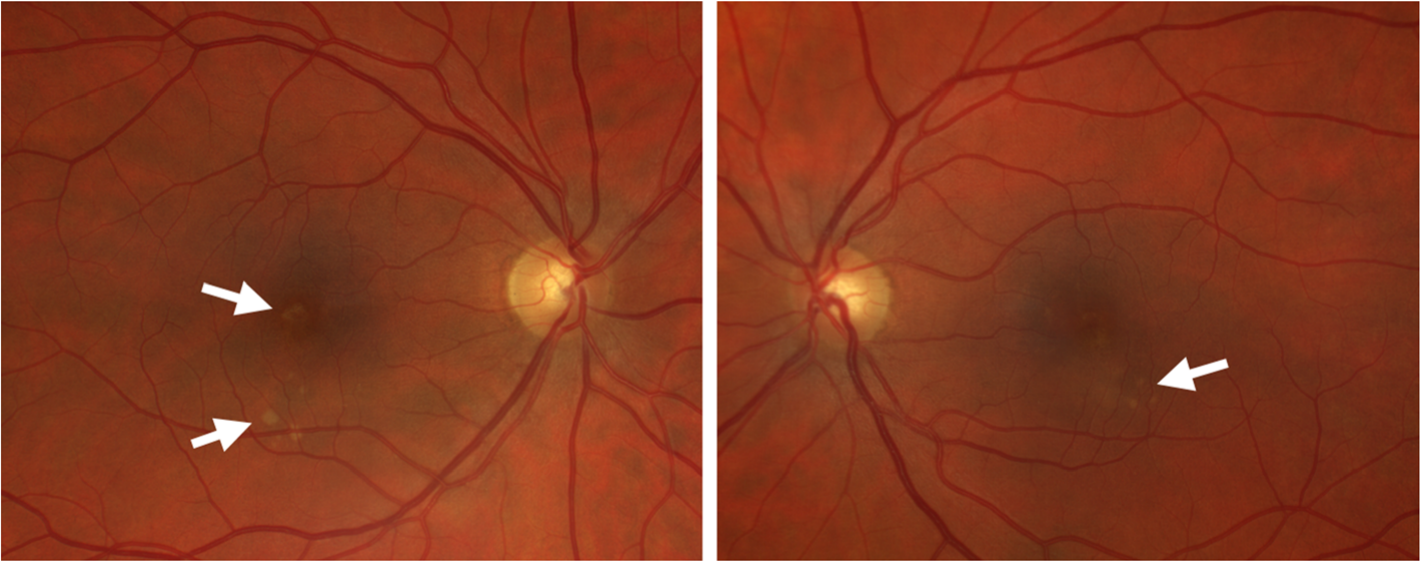
Fundoscopy revealed an attenuation of the foveal reflex with subtle yellow-white subretinal deposits in the inferotemporal part of the macula (white arrows)

**Fig. 2:**
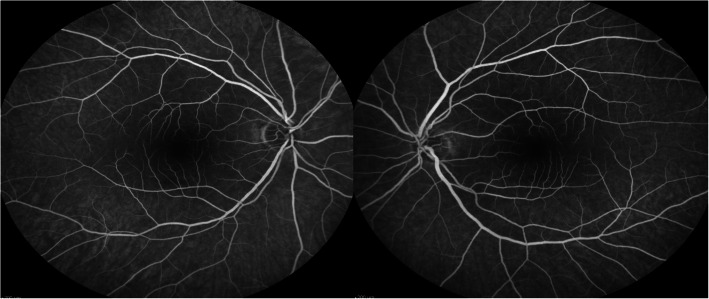
Fluorescein angiography did not show staining or leakage in the mid-phase

**Fig. 3:**
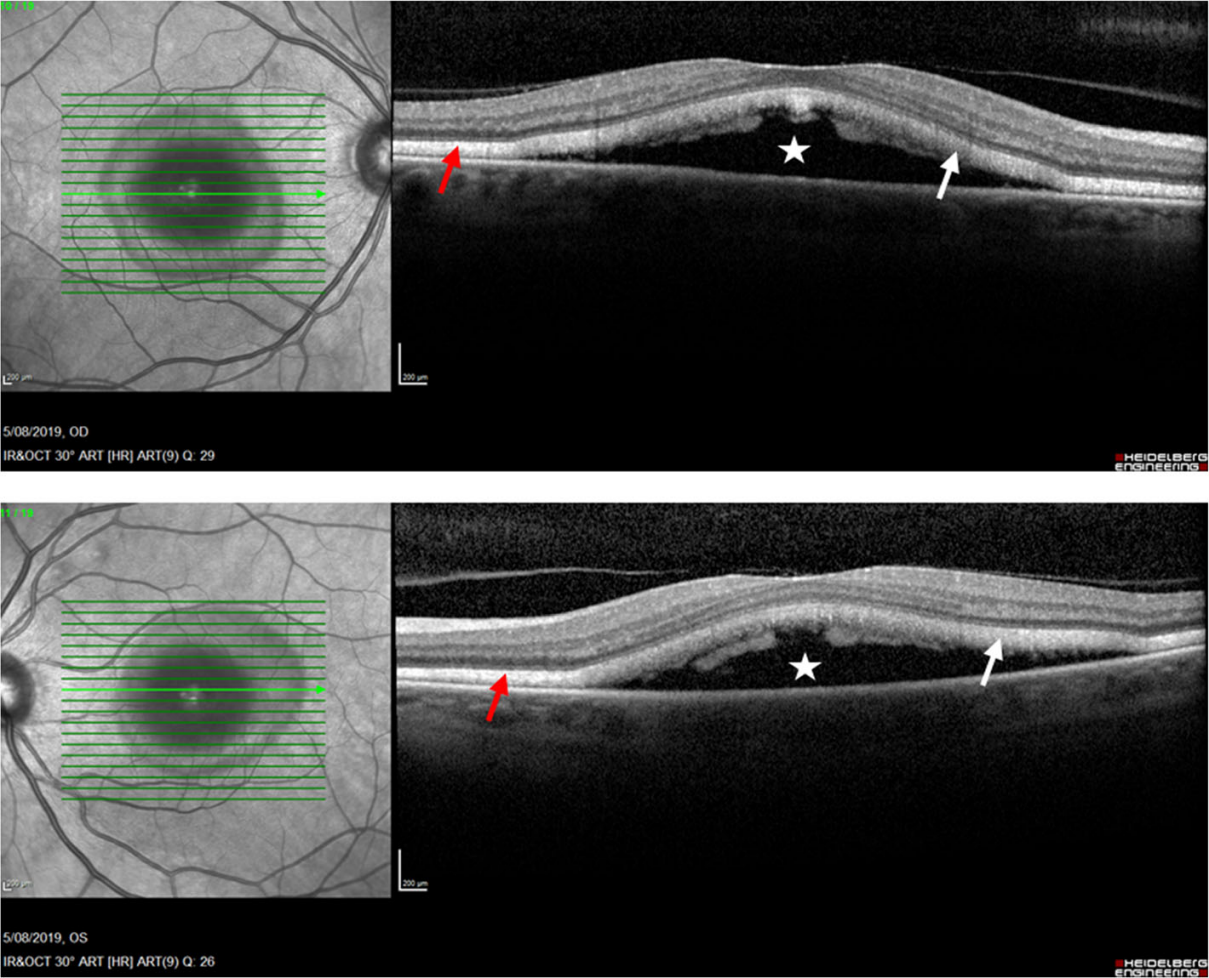
OCT cross-sections illustrated bilateral neurosensory retinal detachment (white star) with a thick, highly reflective band at the outer photoreceptor layer (white arrow). This reflective band is more prominent centrally but extends to regions without obvious neurosensory detachment (red arrow)

**Fig. 4:**
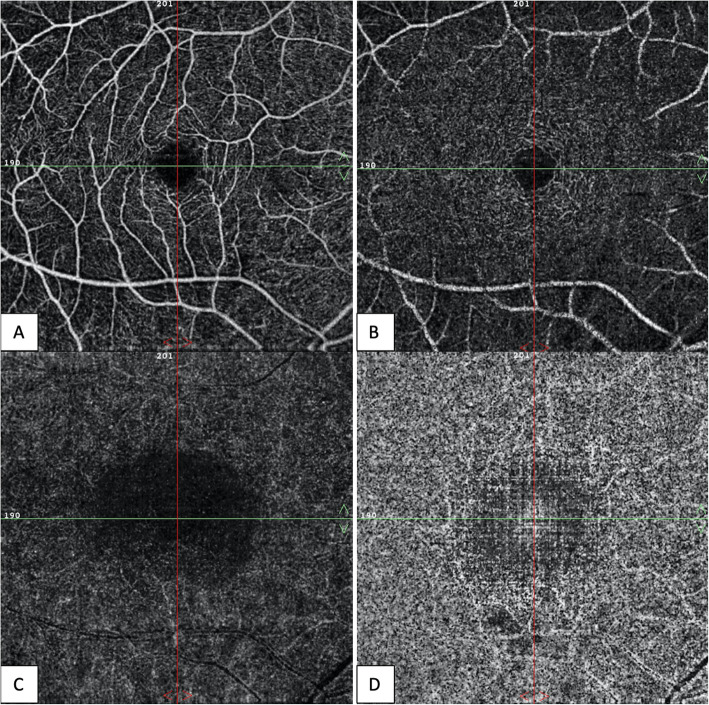
Right eye. On OCT-angiography the superficial capillary plexus (**A**) and deep capillary plexus (**B**) appeared normal. The outer retina (**C**) and choriocapillaris (**D**) both had shadowing optical artifacts from subretinal fluid and projection artifacts from superficial retinal vessels

**Fig. 5:**
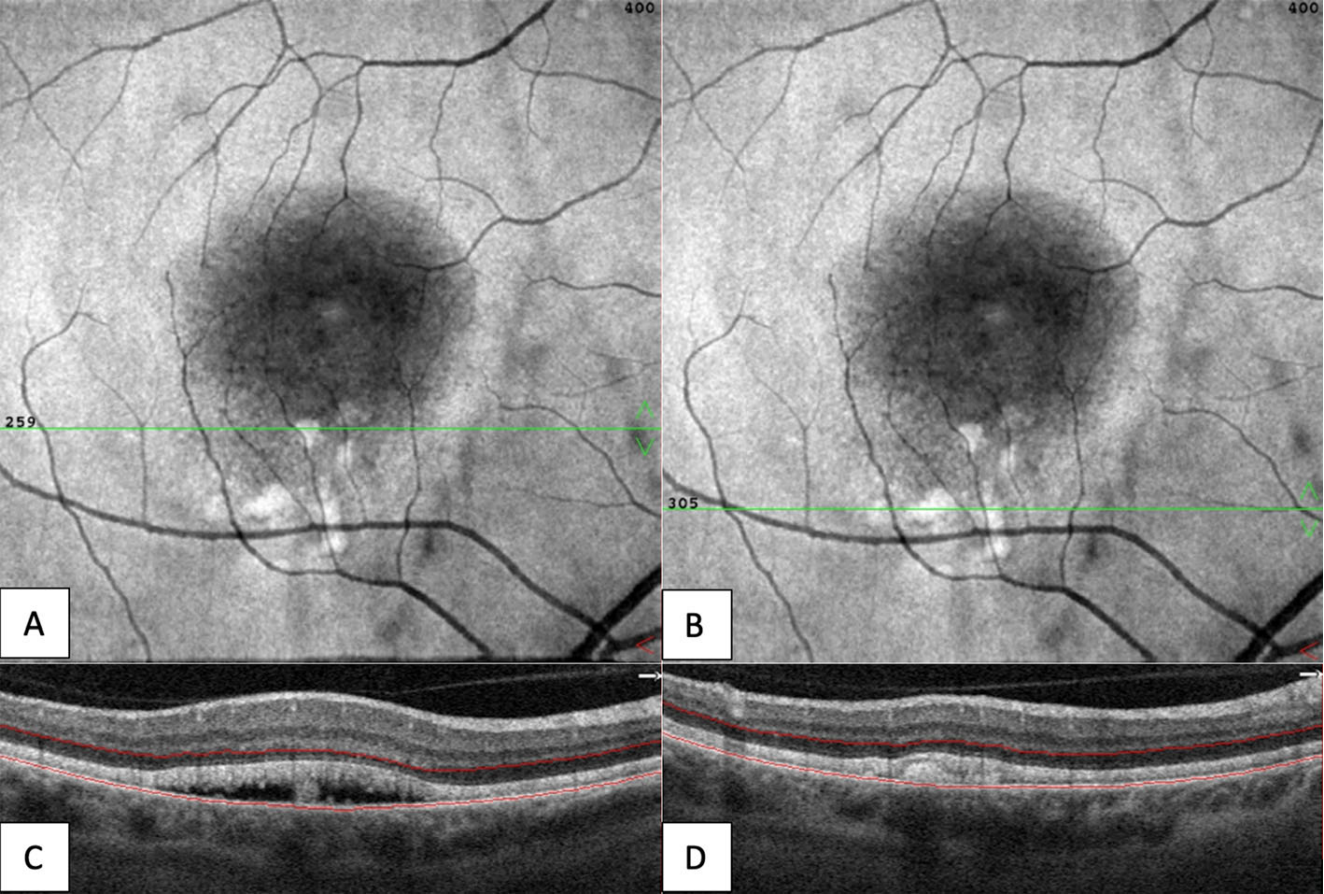
Right eye. En face structural OCT at the level of the photoreceptors (**A**, **B**) showed signal attenuation consistent with subretinal fluid and focal areas of increased signal corresponding to hyperreflective vitelliform material seen on cross-sectional OCT (**C**, **D**). The green line shows the location of OCT B-scan with en face OCT segmentation boundaries (red lines)

A 54-year-old woman was referred by her oncologist because of blurry vision in both eyes. She also complained of a yellow spot in the left eye. The patient had a history of vaginal mucosal melanoma which was diagnosed in 2018. She underwent a total hysterectomy with a partial vaginectomy. In 2019 her gynaecologist discovered a recurrence of the tumor (mucosal melanoma) with multiple metastases in the lungs and lymph nodes (stage IV-M1b). The oncologist started a first-line treatment with pembrolizumab 200 mg intravenously once every three weeks. The first dose was administered the 6th of May 2019. A second dose should have been given the 27th of May but was put on hold due to immune-related thyroiditis. Eventually the second dose was given the 3rd of June. The third and fourth doses were given respectively on the 24th of June and the 15th of July. The ocular symptoms of this patient started after these four treatments with pembrolizumab. Her first visit at the consultation of Ophthalmology was the 5th of August. Her best corrected visual acuity was still 10/10 in both eyes with a correction of + 2.00 bilaterally, though she never wore glasses before. The anterior eye segment and vitreous showed no signs of inflammation. Fundoscopy revealed an attenuated macular reflex with subtle yellow-white subretinal deposits in the inferotemporal part of the macula (shown in Fig. 1). A normal optic disc and periphery were visualized in both eyes. Fluorescein angiography did not show staining or leakage in the mid-phase, nor a late staining (shown in Fig. 2). Spectral-domain OCT (SD-OCT) indicated overall retinal thickening of the central part of the macula in both eyes. Cross-sections illustrated bilateral neurosensory retinal detachment with a thick, highly reflective band at the outer photoreceptor layer. This reflective band is more prominent centrally but extends to regions without obvious neurosensory detachment (shown in Fig. 3). On the OCT-angiography the superficial and deep capillary plexus appeared normal. The outer retina and choriocapillaris both had shadowing optical artifacts from subretinal fluid (shown in Fig. 4). En face structural OCT at the level of the photoreceptors showed signal attenuation consistent with subretinal fluid and focal areas of increased signal corresponding to hyperreflective vitelliform material seen on cross-sectional OCT (shown in Fig. 5). Fundus autofluorescence (FAF) showed an increased autofluorescence most prominent in the inferotemporal part of the macula in both eyes, corresponding to the deposits.

After a multidisciplinary consult with the oncologists the treatment of the patient with pembrolizumab was temporarily interrupted due to the ocular problems, but also because of the manifestation of other immune-related adverse events. The patient showed at the same time symptoms and signs of a sarcoid-like syndrome with prominent mediastinal lymph nodes and grade 1 pneumonitis. Nevertheless, a partial remission of the metastatic melanoma was seen after four administrations of pembrolizumab.

**Fig. 6:**
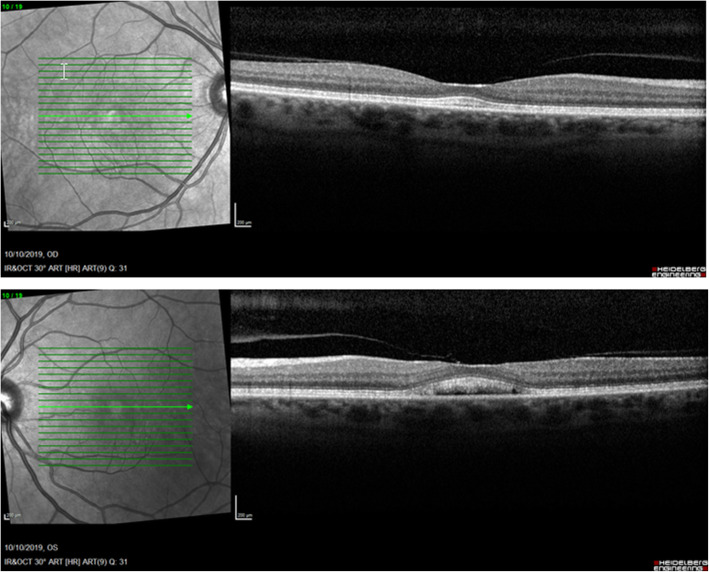
After two months of follow-up (and withdrawal of pembrolizumab) OCT showed no longer subretinal fluid in the right eye, though still a misalignment of the outer retinal layers. In the left eye there was still some residual fluid, however less than before

**Fig. 7:**
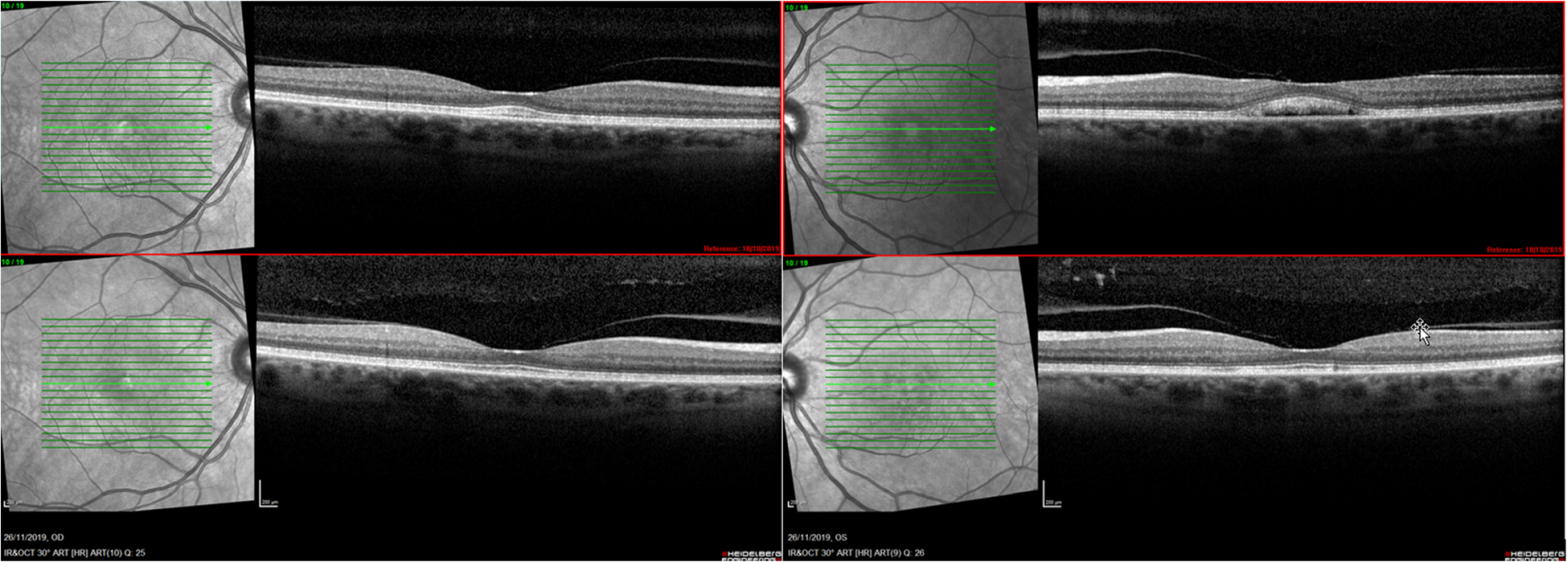
After nearly four months of follow-up (on the 26th of November) a total resolution of the subretinal fluid in both eyes was visualized. This picture illustrates the evolution and spontaneous restoration of the subretinal fluid and misalignment of the outer retinal layers over a period of almost seven weeks

During the following visits we slowly saw an improvement of the neurosensory retinal detachment in both eyes. On the 10th of October, two months after the initial visit, SD-OCT showed no longer subretinal fluid in the right eye, though still a misalignment of the outer retinal layers. In the left eye there was still some residual fluid, however less than before (shown in Fig. 6). After nearly four months of follow-up and with complete discontinuation of pembrolizumab ever since the first visit, we visualized a total resolution of the subretinal fluid in both eyes without the use of systemic or local corticosteroids during this period (shown in Fig. 7). Although the vitelliform lesions remained present. The oncologists also concluded a positive evolution of the sarcoid-like syndrome and the pneumonitis.

## Discussion and conclusions

The patient described in this case presented with multiple vitelliform lesions accompanied by serous retinal detachments and photoreceptor outer segment thickening, all signs of AEPVM, which manifested during pembrolizumab treatment. This case suggests a link between the use of pembrolizumab as a first-line treatment for metastatic recurrent vaginal mucosal melanoma and acute exudative polymorphous vitelliform maculopathy.

The interaction of PD-1 expressed on T cells with its ligand PD-L1 (B7H1) and PD-L2 (B7DC) is known to be a mechanism of T cell inhibition. In 2008 a study was presented by Usui et al. which examined whether human or murine retinal pigment epithelium (RPE) cells express B7H1 and B7DC. The data suggested that PD-L1 is expressed on RPE cells and that this expression plays an immunosuppressive role in ocular inflammation, which may contribute to immune privilege in the posterior segment of the eye [6]. Pembrolizumab inhibits PD-1 binding to PD-L1 allowing T cell activation. This provokes not only an antitumor immune response but may also provoke an immune response against the patient’s own RPE cells with a disturbance in the RPE homeostasis with breakdown of the epithelium barrier and a decrease of the RPE phagocytic function which generates an accumulation of abnormal deposits of lipofuscin and subretinal fluid [3].

In this case pembrolizumab withdrawal appears to have been of great influence on the recovery process of the retina. Corticosteroids administered as topical eyedrops, periocular or intraocular injections as well as a systemic preparation are treatment options to consider when vision and/or the structural integrity of the retina are threatened [4, 5]. However, treatment with corticosteroids seems to be controversial, since visual acuity and/or retinal anatomy often improve spontaneously without treatment, as was the case with our patient. A case report presented by Modi et al. described no improvement of AEPVM in a young man with steroid therapy [7]. The same applies to a more recent report of two cases by Kemels et al. The two patients presented with AEPVM within one month after the initiation of nivolumab. The authors outlined no significant resolution of the neurosensory retinal detachment or the subretinal lipofuscin deposits after administration of systemic or local corticosteroids. In contrast to our case, they concluded that discontinuation of the immunotherapy did not bring significant resolution of the lipofuscin rich subretinal fluid. But in the first case there was a mild decrease of subretinal fluid one month after stopping nivolumab. The patient died three months later. In their second case nivolumab was discontinued for two months, which did not lead to significant resolution of the serous detachment. When nivolumab was re-initiated the patient simultaneously underwent a surgical resection of the primary tumor. This combination was accompanied by a significant reduction of the subretinal fluid [8]. Compared to our case, their shorter immune checkpoint inhibitor free period could partly explain why little improvement was seen. In our case, discontinuation of pembrolizumab treatment resulted in fluid resolution, though more slowly than initially expected. An explanation could be that pembrolizumab initiates a complex cascade of immune reactions, which don’t stop immediately after drug cessation and with longer lasting effects (comparing to MEK inhibitors for example).

There have been many hypotheses of the causes of AEPVM, but most research has shown the origin to be at the level of the RPE where there is an autoimmune retinal pigment epitheliopathy trigger from an infection, a neoplasm, or some other autoimmune process. To date, only a few cases have been reported about AEPVM and the use of immune checkpoint inhibitors in general and even fewer about Pembrolizumab. Miyamoto et al. reported a case describing multiple bilateral serous retinal detachments and outer segment thickening without inflammation in a patient treated with the PD-1 immune checkpoint inhibitor nivolumab [3]. Kemels et al. hypothesized that nivolumab indirectly provoked paraneoplastic AEPVM [8]. Harpal et al. presented a case of a patient with AEPVM during vemurafenib and pembrolizumab treatment for metastatic melanoma. The authors concluded that it is difficult to differentiate between a direct association with vemurafenib and an indirect triggering of an autoimmune paraneoplastic process. They also point out that pembrolizumab might have played a role in the development of AEPVM [1].

The differential diagnosis in our case is whether this vitelliform maculopathy originates from a paraneoplastic reaction, or whether it is a pembrolizumab-induced retinopathy or a combination of both. The latter implies a paraneoplastic induced immune reaction aggravated or triggered by pembrolizumab. The fact that the visual symptoms started soon after the initiation of the immunotherapy and that discontinuation of pembrolizumab leaded to complete resolution of the subretinal fluid points to the use of pembrolizumab as a possible cause of AEPVM rather than a purely paraneoplastic reaction, but this doesn’t exclude the combined hypothesis. Possibly the treatment with pembrolizumab provoked a paraneoplastic AEPVM indirectly by inducing an immune response against the tumor and consequently against the RPE (cross-reaction). Addressing these possibilities is beyond a mere academic research since adjustments of the therapy could be life threatening. Future identification of similar associations and investigation are required to unequivocally link pembrolizumab to AEPVM in metastatic melanoma.

In conclusion, this case suggests a possible causative role of pembrolizumab in the development of acute exudative polymorphous vitelliform maculopathy. Whether there is an underlying paraneoplastic problem remains indeterminate, though there is a strong likelihood. It is clear that checkpoint inhibitors, including pembrolizumab, can induce ocular and orbital immune-related adverse events. Therefore, patients receiving pembrolizumab should undergo a baseline comprehensive eye examination and should at least be counseled to seek medical attention immediately if visual changes occur. A strong cooperation between oncologists and ophthalmologists is essential in the diagnosis and prompt management of these side effects. The increase in the use of immune checkpoint blockade therapies will lead to new clinical insights in the near future.

## Data Availability

Not applicable.

## Abbreviations

AEPVM: Acute exudative polymorphous vitelliform maculopathy
OCT: Optical coherence tomography
PD-1: Programmed cell death receptor-1
PD-L1: Programmed cell death protein ligand-1
IRAEs: Immune-related adverse events
SD-OCT: Spectral-domain OCT
FAF: Fundus autofluorescence
RPE: Retinal pigment epithelium
